# Is there a specific calcium signal out there to decode combined biotic stress and temperature elevation?

**DOI:** 10.3389/fpls.2022.1004406

**Published:** 2022-11-03

**Authors:** Sarah Carpentier, Didier Aldon, Richard Berthomé, Jean-Philippe Galaud

**Affiliations:** ^1^ Laboratoire de Recherche en Sciences Végétales, Université de Toulouse, CNRS, UPS, Toulouse, France; ^2^ Laboratoire de Recherche en Sciences Végétales, Université de Toulouse, CNRS, UPS, Toulouse INP, Auzeville-Tolosane, France

**Keywords:** biotic stress, calcium channels, calcium sensors, calcium signaling, combined stress, temperature elevation, thermosensors

## Introduction

Because of their motionless state, plants are exposed in their environment, often simultaneously, to multiple biotic (bacteria, fungi, oomycetes, viruses, insects) and abiotic (temperature, drought, water and nutrient availability) constraints. To adapt, plants have developed amazing abilities allowing the perception of their situation and the establishment of specific responses (Velásquez et al., 2018; Delplace et al., 2022). These capacities are even more important in the context of climate change for which various scenarios forecast, for the coming decades, an increase in the duration, intensity and frequency of extreme weather events, emergence of new pathogens and/or geographical expansion of their distribution and an increase in epidemics (McDonald & Stukenbrock, 2016; Chaloner et al., 2021; IPCC, 2021). Among climatic parameters, temperature is expected to fluctuate the most by the end of the century (IPCC, 2021). Worryingly, a growing number of studies describe that an elevation in ambient temperature has a negative impact on the majority of the resistance mechanisms deployed by plants (Desaint et al., 2021). It is therefore crucial to understand the effects of pathogens combined with elevated temperature on plant responses and to identify the mechanisms that contribute to robustness of resistance. However, our knowledge on the strategies developed by plants to face the combined constraints remains fragmented. Except for system involving the Arabidopsis plant model, the impact of temperature elevation combined with the induction of plant immunity through flagellin (flg22) treatment have mostly been investigated at the transcriptomic level (Huot et al., 2017; Velásquez et al., 2018; Kim et al., 2022; Yuan and Poovaiah, 2022). Indeed, immune mechanisms, recently demonstrate to be much more interconnected than the simplified “zig-zag model” previously proposed more than a decade ago, restrict pathogen proliferation (Jones and Dangl, 2006; Ngou et al., 2021; Yuan et al., 2021a). Early stages of perception and signaling are often decisive to elicit appropriate responses. Most external stimuli induce an increase in cytosolic calcium concentration that is central to initiate later responses (Liu et al., 2020). In eukaryotes, intracellular calcium variations are considered a universal second messenger, which must be decoded and relayed by calcium sensors to downstream targets allowing the implementation of finely-tuned, spatio-temporal responses, including transcriptome modifications (Ranty et al., 2016; Aldon et al., 2018; Liu et al., 2020; Luan and Wang, 2021). Numerous studies indicate that calcium sensors are involved in many processes such as plant development, biotic and abiotic responses (Zhu et al., 2015; Luan and Wang, 2021). Therefore, this opinion paper aims at providing evidence supporting the premise that proteins participating to the generation and decoding of calcium variations, would be central in shaping plant responses against combined biotic and elevated temperature constraints.

### Calcium signaling in plant immunity

**Figure 1:**
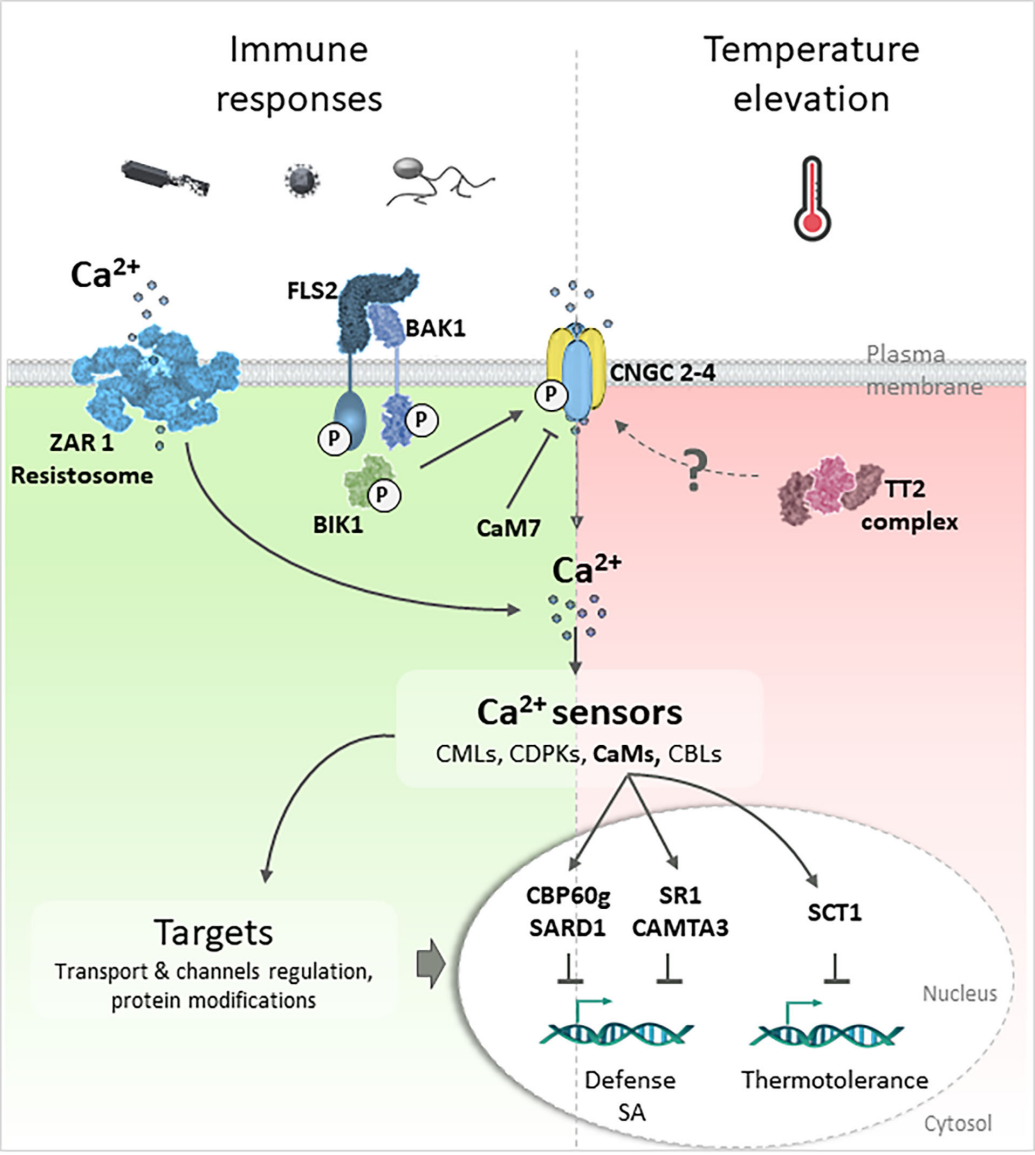
Involvement of calcium signaling in response to biotic and elevated temperature constraints. Plant defense responses to pathogens trigger calcium variations which are relayed by calcium sensors (CaM, CMLs, CDPKs or CBLs) to targets allowing the establishment of specific responses. The *P. syringae*’s flagellin is recognized by the Pathogen Recognition Receptors (PRR) complex FLS2/BAK1 and induce PTI leading to the kinase BIK1 phosphorylation which phosphorylates CNGC calcium channels and allows calcium entry. The NLR ZAR1 recognizes *P. syringae* effectors delivered in the cytosol inducing ETI and forms a pentameric structure integrating the plasmalemma (resistosome) (Bi et al., 2021) to act as a calcium-permeable channel. CNGC2-4 are also temperature-sensitive channels allowing calcium entry upon temperature elevation (Guihur et al., 2022b). Their activity is negatively regulated by the CaM7 (Tian et al., 2019). In response to elevated temperature, the rice TT2 complex, composed by a G-subunit, is involved in thermotolerance (Kan et al., 2022). TT2 signaling pathway is mediated by the transcription factor SCT1 that interact with CaM to repress genes required for thermotolerance. Under combined flagellin treatment and elevated temperature condition, SA production is repressed and PTI inhibited through CBP60g/SARD1 and CaM-binding SR1/CAMTA3 dependent pathways (Huot et al., 2017; Kim et al., 2022).

The generation of intracellular calcium variation in response to pests or PAMPs has been described for many years and the contribution of calcium signaling in the establishment of resistance to pathogens is supported by many studies (Atkinson et al., 1990; Knight et al., 1991; Blume et al., 2000; Lecourieux et al., 2006). To date, genetic approaches clearly established that the channels and pumps involved in calcium signal generation and dissipation as well as calcium sensors, contribute to the implementation of efficient responses against pathogens. Until 2019, the nature of calcium channels involved in immune responses remained largely unknown. Tian et al. (2019) and Wang et al. (2019) showed, in *Arabidopsis* and rice respectively, that Cyclic Nucleotide-Gated Channels (CNGCs), which are calcium permeable channels, are crucial for calcium increase upon flg22 or chitin perception. The perception of flg22 by the FLAGELLIN-SENSITIVE 2 (FLS2)/BRI1-ASSOCIATED RECEPTOR KINASE (BAK1) complex induce Pathogen Associated Molecular Patterns (PAMP)-Triggered Immunity (PTI) by leading to the phosphorylation and activation of the CNGC2/CNGC4 heterodimer by the BOTRYTIS-INDUCED KINASE1 (BIK1) (Tian et al., 2019) (**Figure 1**). CNGC’s activity is also regulated by calmodulin (CaM), as in the case of CaM7 in Arabidopsis, which prevents CNGC2/4 activity and calcium influx (Tian et al., 2019) (**Figure 1**). More recently, HOPZ-ACTIVATED RESISTANCE 1 (ZAR1), an intracellular Nucleotide-binding Leucine -rich Receptor (NLR), was shown to interact with bacterial effectors inducing Effector-Triggered Immunity (ETI) and to form pentameric complexes *in vitro* with protein kinases called resistosomes (Bi et al., 2021). This complex acts as a cation-selective channel engaged in the plasma membrane that induces calcium influx, reactive oxygen species production, cell death and finally immunity to plants (Bi et al., 2021) (**Figure 1**). Usually seen as two independent steps in plant immunity, PTI and ETI have been recently demonstrated to potentiate one another (Ngou et al., 2021; Yuan et al., 2021a) and calcium signaling is known to be a key player in both of them (Yuan et al., 2021b; Köster et al., 2022). In addition to the identification of the previous channels, strong evidence implicating CaM; the Calmodulin-like proteins (CMLs) (Zhu et al., 2015), Calcium-Dependent Protein Kinases (CDPKs) (Shi et al., 2018; Bredow and Monaghan, 2019) and Calcineurin B-Like (CBLs) (Ma et al., 2020; Tang et al., 2020) support that calcium sensors are essential in biotic stress responses (**Figure 1**). Increasing data report the contribution of CMLs to defense regulation against various pathogens such as oomycetes, bacteria or insects as shown for CML8, which contributes to defense against multiple pathogens varying in lifestyles and infection mode in Arabidopsis (Zhu et al., 2017; Zhu et al., 2021). In tomato, the silencing of CML55 suppresses infection by *Phytophthora capsici* (Zhang et al., 2022) whereas the silencing of CML13 in pepper enhances the plants’ susceptibility to *Ralstonia solanacerarum* (Shen et al., 2020). A. Mithöfer’s group also illustrated the contrasting effect of different CMLs in the plant-herbivore interaction. Loss of function of CML42 in Arabidopsis enhances resistance to the caterpillar *Spodoptora littoralis*, which correlates with the induction of jasmonic acid-responsive genes and to an accumulation of glucosinolates (Vadassery et al., 2012). In contrast, mutation of CML37 increases Arabidopsis susceptibility to this herbivore (Scholz et al., 2014). Other examples show contributions of CMLs in response to both biotic or abiotic constraints, but so far this has not been studied under combined stress conditions. For example, CML24 is a multifunctional CML involved in biotic and abiotic responses as well as in developmental processes (Delk et al., 2005; Tsai et al., 2007; Ma et al., 2008; Zhu et al., 2022) and CML37, described as a regulator of plant responses to *S. littoralis*, also positively contributes to drought responses in Arabidopsis (Scholz et al., 2015). Interestingly, CML37 and 42 also act antagonistically when plants are exposed to water deficit, herbivores or necrotrophic fungi (Heyer et al., 2021). Other CMLs may also contribute differently to the plant response depending on the constraint such as the Arabidopsis CML9 which is involved in both biotic and abiotic processes. CML9 loss of function results in a higher susceptibility to *P. syringae* (Leba et al., 2012) and to a lower sensitivity to water deficit (Magnan et al., 2008). Although this opinion paper mostly presents case studies on CMLs, excellent reviews highlight the importance of other calcium sensors, such as CDPKs and CBLs, in the calcium decoding steps related to plant immunity or responses to abiotic constraints and developmental processes (Bredow and Monaghan, 2019; Yip Delormel and Boudsocq, 2019; Ma et al., 2020; Tang et al., 2020).

### Calcium signaling in heat stress

Collectively, calcium signaling and decoding appear to significantly contribute to plant immunity and abiotic constraints (Patra et al., 2021). Regarding stress associated with temperature elevations, Gong et al. (1998) reported that heat-shock induces transient increases in calcium concentration and that exogenous calcium supply to tobacco seedlings promoted plant tolerance to subsequent transitory heat treatments, whereas the use of a calcium chelator had the opposite effect. It was therefore hypothesized that calcium signaling could significantly contribute to the thermotolerance of plants. Interestingly, the chloroplast has emerged during the last ten years (Navazio et al., 2020) to be important in calcium signaling at elevated temperatures as transient temperature elevations to 40°C were demonstrated to increase free calcium in the stroma but not in the cytosol (Lenzoni and Knight, 2019). Genetic approaches indicate that this calcium increase depends on the thylakoid Calcium-sensing receptor (CAS), already proposed to decode the calcium signal following perception of PAMPs (Nomura et al., 2012). Moreover, Finka et al. (2012) reported that two calcium channels, CNGCb from moss and CNGC2, its ortholog in Arabidopsis, act as thermosensors in plants. Disruption of CNGCb or CNGC2 produced a hyper-thermosensitive phenotype. Upon temperature elevation, the CNGC2/CNGC4 complex opens at the plasmalemma allowing the entry of calcium (Guihur et al., 2022b) (**Figure 1**). This complex shares features (localization, temperature increase opening, control of external calcium entry) with the animal thermosensor channel TRPV1 whose discovery was recognized by the award of the Nobel Prize in 2021 (Julius, 2013; Kefauver et al., 2020; Guihur et al., 2022a). Moreover, the recent identification of the *Thermotolerance 2 (TT2)* locus in rice encoding a G-subunit responsible for thermotolerance brings into light, albeit indirectly, the importance of calcium signaling in the plant response to temperature fluctuation (Kan et al., 2022). Indeed, among the downstream events linked to TT2, the sensing calcium transcription factor 1 (SCT1) interacts with calmodulin to repress target genes required for thermotolerance.

### Calcium signaling actors in combined stress?

Recently, 14 common QTLs were identified among the 42 and 43 QTLs underlying the rice response to biotic or heat constraints respectively. Meta-expression analysis of the 1265 genes underlying the 14 QTLs showed that 24 genes were involved in calcium signaling, supporting its importance in plant response to both constraints (Kumar et al., 2022). However, common QTLs identified with constraints applied individually do not necessarily imply that they would also be detected when applying these constraints in combination. Indeed, transcriptome analyses, which constitute the majority of studies developed to understand the processes involved in the plant response to constraints applied in combination, showed that the plant response cannot simply result from the addition of mechanisms involved in responding to the same constraints applied individually (Suzuki et al., 2014; Pandey et al., 2015; Desaint et al., 2021). When constraints are applied in combination, few common genes are identified, their expression may be modulated differently (Rasmussen et al., 2013; Farjad et al., 2018) and depending on the severity or order of the applied constraints (Onaga et al., 2017). To date, there are only a few studies suggesting calcium signaling involvement in plant responses to combined constraints. Among these, Cao et al. (2017) showed that application of PAMPs together with salt altered calcium signal generation with an additive effect compared to the individual treatments. However, while the authors suggested this could result from activation of different calcium channels, it cannot be ruled out that this may also be associated with transcriptome changes or post-translational modifications of proteins involved in the formation of calcium-permeable channels, resulting in changed channel activity or other processes such as calcium release from other cellular compartments.

Recent studies present more convincing data that remains difficult to generalize, as they were obtained for a specific system implicating the Arabidopsis response to the combination of flg22 treatment and temperature elevation. Huot et al. (2017) showed a temperature elevation from 21°C to 28°C and 33°C increases the plants’ susceptibly and compromises PTI. Through transcriptome analysis, they proposed that the increase in plant susceptibility is associated with suppression of salicylic acid (SA) production. This inhibition appears to be independent of temperature-sensitive signaling pathways involving phytochrome B, the transcription regulators EARLY FLOWERING 3 and PHYTOCHROME INTERACTING FACTOR 4, which were previously proposed to participate in the integration of the effect of temperature on the plant and the coordination between growth and immunity (Gangappa and Kumar, 2018). In this case, it has been proposed the modulation of SA-mediated immunity relies on the CaM-binding transcription factor (TF) SR1/CAMTA3 dependent pathways, indirectly suggesting the contribution of calcium signaling (Huot et al., 2017) (**Figure 1**). These results, although very interesting, are surprising, since SR1/CAMTA3 is known to suppress plant immunity and promote plant development in non-stressful environments (Yuan et al., 2018). Its involvement in plant response to combined constraints therefore suggests different modes of action or regulation of this TF, such as putative modifications in its phosphorylation status. These results also support the central role of calcium in the combined responses to *P. syringae* and elevated temperature through suppression of SA production at 28°C. Indeed, Kim et al. (2022) demonstrated the involvement of the Calmodulin Binding Protein 60g (CBP60g) and Systemic Acquired Resistance Deficient 1 (SARD1) TF, already known to participate in PAMP-induced SA accumulation and in PTI response to *P. syringae* (Wang et al., 2011) (**Figure 1**).

## Conclusions

To date, most research on plant stress physiology has been conducted by applying biotic or abiotic stresses separately, in simplified systems, under controlled conditions. By contrast, the impact of combined constraints is alarmingly poorly understood. Few studies have explored the contribution of calcium in plant responses to such condition but some results obtained by infecting plants at elevated temperature strongly support its central role in signaling following perception of multiple biotic and abiotic constraints. The scope of possible functions of calcium may arise from several molecular processes or actors such as CNGCs as calcium channels and thermosensors or calcium sensors. However, the associated mechanisms controlling its concentration fluctuation, subcellular accumulation and signaling remain to be deciphered. Therefore, understanding how calcium acts at the crossroad of biotic and abiotic constraints is relevant. Further information could be obtained by using *a priori* approaches (i.e genetics, comparative transcriptomics) and functional analyses of calcium toolbox players involved in combined stresses. Alternatively, genome-wide association mapping (GWA) analyses could uncover candidates, through the identification of the genetic basis underlying the natural diversity of the plants’ response to combined biotic and abiotic constraints. Indeed, such a strategy has already demonstrated its potential to identify genes underlying quantitative resistance to different pathogens (French et al., 2016; Bartoli and Roux, 2017) and in the response of plants to pathogens and temperature elevation (Aoun et al., 2017; Aoun et al., 2020; Bruessow et al., 2021). Such knowledge could provide solutions for plant breeding in order to preserve the agronomic and economic performance of crop species endangered by global change.

## Author contributions

All authors listed have made a substantial, direct, and intellectual contribution to the work and approved it for publication.

## Funding

SC is the recipient of a fellowship from the “École Universitaire de Recherche (EUR)” TULIP-GS (ANR-18-EURE-0019) and the Region Occitanie, Pyrénées-Méditerranée. This work was supported by the Université Paul Sabatier (Toulouse, France), the CNRS (France), the INRAE and by the Agence Nationale de la Recherche (ANR) (ANR-17-CE20-0017-01) thanks to the CaPPTure project. This study is set within the framework of the “Laboratoires d’Excellences (LABEX)” TULIP (ANR-10-LABX-41). 

## Acknowledgments

We thank Julie Cullimore for her comments on the manuscript and proofreading of the English text.

## Conflict of interest

The authors declare that the research was conducted in the absence of any commercial or financial relationships that could be construed as a potential conflict of interest.

## Publisher’s note

All claims expressed in this article are solely those of the authors and do not necessarily represent those of their affiliated organizations, or those of the publisher, the editors and the reviewers. Any product that may be evaluated in this article, or claim that may be made by its manufacturer, is not guaranteed or endorsed by the publisher.
